# Daily volume of cases in emergency call centers: construction and validation of a predictive model

**DOI:** 10.1186/s13049-017-0430-9

**Published:** 2017-08-29

**Authors:** Damien Viglino, Aurelien Vesin, Stephane Ruckly, Xavier Morelli, Rémi Slama, Guillaume Debaty, Vincent Danel, Maxime Maignan, Jean-François Timsit

**Affiliations:** 1 University Grenoble Alps, Emergency Department and Mobile Intensive Care Unit, CHU Grenoble Alps, Grenoble, France; 2 University Grenoble Alps, INSERM U823, Institut Albert BONNIOT, Grenoble, France; 3 University Grenoble Alps, CNRS UMR 5525, TIMC-IMAG laboratory, Team PRETA, Grenoble, France; 4 Paris Diderot University, Medical and Infectious Intensive Care Unit, Hôpital Bichat Claude Bernard, AP-HP, Paris, France

**Keywords:** Emergency medical services, Health service needs and demand/trends, Models, Theoretical, Safety management/methods

## Abstract

**Background:**

Variations in the activity of emergency dispatch centers are an obstacle to the rationalization of resource allocation. Many explanatory factors are well known, available in advance and could predict the volume of emergency cases. Our objective was to develop and evaluate the performance of a predictive model of daily call center activity.

**Methods:**

A retrospective survey was conducted on all cases from 2005 to 2011 in a large medical emergency call center (1,296,153 cases). A generalized additive model of daily cases was calibrated on data from 2005 to 2008 (1461 days, development sample) and applied to the prediction of days from 2009 to 2011 (1095 days, validation sample). Seventeen calendar and epidemiological variables and a periodic function for seasonality were included in the model.

**Results:**

The average number of cases per day was 507 (95% confidence interval: 500 to 514) (range, 286 to 1251). Factors significantly associated with increased case volume were the annual increase, weekend days, public holidays, regional incidence of influenza in the previous week and regional incidence of gastroenteritis in the previous week. The adjusted R for the model was 0.89 in the calibration sample. The model predicted the actual number of cases within ± 100 for 90.5% of the days, with an average error of −13 cases (95% CI: -17 to 8).

**Conclusions:**

A large proportion of the variability of the medical emergency call center’s case volume can be predicted using readily available covariates.

**Electronic supplementary material:**

The online version of this article (10.1186/s13049-017-0430-9) contains supplementary material, which is available to authorized users.

## Background

Emergency dispatch centers are today a key component of emergency care. They receive calls requiring an assessment and emergency assistance is dispatched if necessary. Call centers mobilize a large number of personnel and technical resources. The number of calls treated in the United States is 240 million per year [1] and is continuously increasing [1, 2]. Adaptations to this increase in activity are currently based on the better organization of centers receiving emergency hotlines: procedures for answering and prioritizing calls have been set up, performance indicators are followed in real time, and additional personnel are planned in case of overload or a catastrophic event [3, 4]. Current budget restrictions require emergency services to meet the challenge of increasing activity with fixed numbers of personnel [5].

Simultaneous with the annual increase in activity, emergency call centers must deal with substantial daily and seasonal variations that may seem highly unpredictable. Some factors influencing these variations have already been identified in order to adapt the size of these services to their annual activity [6]. Beyond the annual trend toward increasing activity, other known factors are social and demographic [6, 7] or related to certain events such as heat waves [8, 9] or cyclones [10]. The effect of these occasional events has been most widely studied for admissions to hospital emergency departments. In contrast, factors related to the day of the week and time of day [11], seasonal or yearly variations [12–14], weather or epidemiological factors [15–17] are rarely taken into account when predicting the activity of emergency call centers. To our knowledge, there have been no studies seeking to predict the overall day-to-day activity as related to these factors. Taking these factors into account could make it possible to estimate the level of demand and better meet needs by rationalizing the internal organization of these services, thus improving the efficiency of emergency care.

The objective of this study was to construct and evaluate the reliability of a prediction model of daily case volume in an emergency call center.

## Methods

### Study design and setting

We split the study period (2005-2011) into a development phase and a validation phases. A model predicting the daily number of cases was developed on the data collected over 4 consecutive years (development period, 2005–2008, 1461 days) in a large emergency call center. The model was applied and its predictive ability tested over the following three-year period (test period, 2009–2011, 1095 days). We considered explanatory variables available at least 1 week before the day to predict and a periodical function to control for the activity’s seasonality. Only data relative to the center’s activity were used, without individual medical information so that the study required no approval by the local ethics committee, according to French legislation.

### Selection of population

From the anonymized case files treated by the emergency call center of a university hospital (SAMU, Service d’Aide Médicale d’Urgence, centre 15, Isère *department*, Grenoble, south-eastern France. Table 1), we computed the total number of cases per day. Each call is first answered by an operator who identifies the request and notes practical information, and then is transferred to a physician-dispatcher. The call center’s automatic computerized system, which records time and date, guaranteed that no calls were missed for the study period. A single event treated by the call center was considered as one case but it could include several successive calls and several responses. Calls for a nonmedical motive were discarded (police, requests for firefighters with no victim involved, erroneous calls), as well as calls concerning inter-hospital transport. The zone covered had a population of 1.34 million inhabitants in 2010, including urban, semi-rural, and mountain populations, with a strong temporal variability related to recreational activities (winter and mountain sports) (Table 1).

**Table 1:** Characteristics of the considered EMS in 2010, according to the consensus-based template (Krüger et al. SJTREM 2011) [18]

Characteristics		Comments
Population covered	1,340,000	
Service area provision	7431.44 km^2^	Heterogeneous; includes relatively flat areas, valleys, mountains and a central urban area
Population density	165 /km^2^	
Operating hours	Full-time	
Emergency call center Resources available		
Physicians	2–5	according to time and day of week
Operators	3–7	according to time and day of week
Dispatchers	Physicians	Attending emergency physicians
Dispatch system	Integrated	Integrated medical emergency communication center
Activation criteria/protocol	Consultation	No criteria, activation after consultation with physician
Calls received per year	480,000	
QS60	92 to 98%	Percentage of calls answered within 60 s
Prehospital care Resources available		
First-responder ambulances	45 to 70	Only ground ambulances
Mobile Intensive Care Units Units	5 to 8	Ground ambulances and two helicopters
First-responder missions /y	45,000	
MICU missions per year	8500	

*QS60* Quality Service 60 s, *MICU* mobile intensive care unit. Adapted from Krüger et al., Scandinavian Journal of Trauma, Resuscitation and Emergency Medicine, 2011

### Description of external variables

We considered epidemiological explanatory factors (the regional incidence of influenza and acute gastroenteritis) published every week by InVS (Institut de Veille Sanitaire) [19, 20]. This epidemiological monitoring is carried out by a network of general practitioners who report the number of cases seen every day. School vacation periods for the different geographic zones (the Grenoble catchment area and the Parisian conurbation) were those notified by the French Ministry of Education.

### Model construction

A linear generalized additive model (GAM) [21–23] of the volume of cases per day was constructed, taking into account the activity’s seasonality, the long-term trend, the day of the week (dummy variables), school vacations (different dummy variable for each holiday period), public holidays, and the regional incidence rates of influenza and acute gastroenteritis (number of cases per 100,000 inhabitants the previous week). Variables to test were chosen based on data availability and a priori hypotheses. The model’s predictive goal required that all the variables included in the model to predict case volume on a given day be available in advance. Thus, the epidemiological data taken into account by the model were those of the week preceeding the days for which case volume would be predicted. The linear relation of each quantitative explanatory variable with the number of cases was checked graphically.

**Fig. 1:**
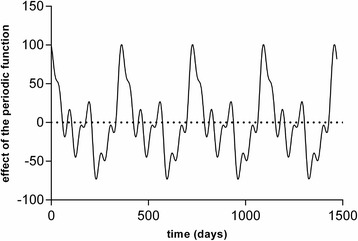
Periodic (yearly) function used to adjust the daily activity to the period of the year (seasonality)

Seasonality [24, 25] was controlled in the model using a periodic function obtained by Fourier decomposition of the signal of the first 4 years (Fig. 1 and Additional file 1). The long-term trend was accounted for by a “year” variable that extrapolated the trend observed. Certain variables were retained in the model independently of their significance, because of their logical relation with other important variables in the model. The variables retained thus formed a coherent group of explanatory factors, such as days of the week or school vacation periods.

### Data analysis

#### Quality of the model

Several methods were used to assess the quality of the models during the development/calibration period (2005–2008) and to evaluate the level of variability: calculation of the determination coefficient (R) and of the adjusted determination coefficient; graphic analysis of the residuals (number of cases observed – number of cases predicted); normality of the residuals using a diagram with the density curve, a quantile/quantile diagram, and using the Kolmogorov-Smirnov test; the White homoscedasticity test; and the Durbin-Watson autocorrelation test.

#### Model performance

The calibrated model (Additional file 3) was applied to the 2009–2011 period (validation cohort), without modification, in order to assess its performance on new data (not used to calibrate it) in a real prediction. The prediction model’s performance for this period was assessed through its ability to predict the number of cases per day within ±100 cases, under which the prediction was deemed reliable. This cut-off of 100 cases corresponds to the minimum number of cases treated per operator per working day. Any variation of less than100 cases should therefore not modify the center’s daily needs in terms of personnel. The prediction capacity for the high and low activity days was also determined. Days with less than the average number of cases observed in the calibration period −100 were considered as “low activity”. Days above the average number of cases observed in the calibration period +100 were considered as “high activity”. On the advice of the reviewers, we a posteriori compared the performance of our complex model to a simpler model that takesinto account only the average of the number of cases each day of the same week during the previous year, plus a coefficient to allow for the augmentation in the annual number of cases.

All the analyses were carried out with SAS 9 software (SAS Institute Inc., Cary, NC, USA) and R (R Foundation, Vienna, Austria).

## Results

### Characteristics of study population

From January 1, 2005 to December 31, 2011, 1,296,153 cases were treated by the emergency call center. The mean number of cases per day during this period was 507 (95% confidence interval, 95%CI, 500 to 514; range, 286 to 1251). The annual number of cases increased by 15.6% yearly. The mean number of ambulance interventions per day was 123 (95%CI, 121 to 124) (range, 64 to 195). A weak correlation was observed between the number of cases per day and the number of ambulance interventions (*R* = 0.31).

### Choice of prediction model

The final model selected for predicting the number of cases (Table 2 and Additional files 1, 2 and 3) included 17 variables: the periodic function, year (long-term trend), six variables corresponding to the days of the week, holidays (coded as the five periods of school vacations in the study zone and the school vacation period for Paris region inhabitants) and the regional incidence rate of flu and acute gastroenteritis in the preceding week. The factors that were significantly related to an increase in call center case volume, after adjustment for the other variables were year (long-term trend) Sundays; Saturdays; public holidays; Christmas vacation; the regional incidence rate of influenza for the preceding week, and the regional incidence rate of acute gastroenteritis of the preceding week. The factors significantly related to a decrease in call center case volume were Tuesdays and spring break vacation. The variables not retained were the weeks of the year, the regional incidence rate of chicken pox, the local pollen levels, and the school vacation periods of the other regions of France not including the study zone or Paris area. These variables were nonsignificant for the number of cases, and t heterogeneity in the pollen levels make it impossible to interpret the results.

**Table 2:** Activity prediction variables model for 2005–2009

Parameter	Estimate^a^	95% CI
Periodic function	0.55	NA
Year (long-term trend)	+ 7.10	(4.36 to 9.85)
Day of the week (Friday^b^ is reference)		
Monday	− 3.02	(− 14.16 to 8.13)
Tuesday	− 14.62	(− 25.71 to − 3.54)
Wednesday	− 5.75	(− 16.83 to 5.35)
Thursday	+ 1.96	(− 9.14 to 13.07)
Saturday	+ 251.75	(240.64 to 262.86)
Sunday	+ 376.07	(364.96 to 387.19)
School and public holidays		
Public holiday	+ 295.03	(277.18 to 312.87)
Christmas vacation	+ 89.05	(72.16 to 105.94)
Winter vacation	+ 12.10	(− 6.25 to 30.46)
Autumn vacation	− 3.81	(− 20.00 to 12.39)
Summer vacation	− 4.44	(− 13.12 to 4.24)
Winter vacation (Paris area)	− 8.23	(− 24.26 to 7.81)
Spring vacation	− 15.83	(− 30.09 to − 1.57)
Epidemics		
Influenza incidence (within 100 ± cases / 100,000 inhabitants the previous week)^c^	+ 5.76	(4.08 to 7.45)
Gastroenteritis incidence (within ± 100 cases /100,000 inhabitants the previous week)^c^	+ 4.74	(0.32 to 9.16)

^a^Represents the number of cases independently attributable to the variable in multivariate analysis

^b^Friday was chosen as a reference because it is the median day in terms of activity

^c^in the local administrative area, called a “*département”* in France, about the size of a county

The model’s coefficient of determination (R) during the development years was 0.889, and the adjusted coefficient of determination (after taking into account the presence of 17 variables and 1461 days) was 0.888. The other tests of the model (normality of residuals, homoscedasticity, and autocorrelation) satisfied the quality criteria.

### Main results (model validation and performance)

**Fig. 2:**
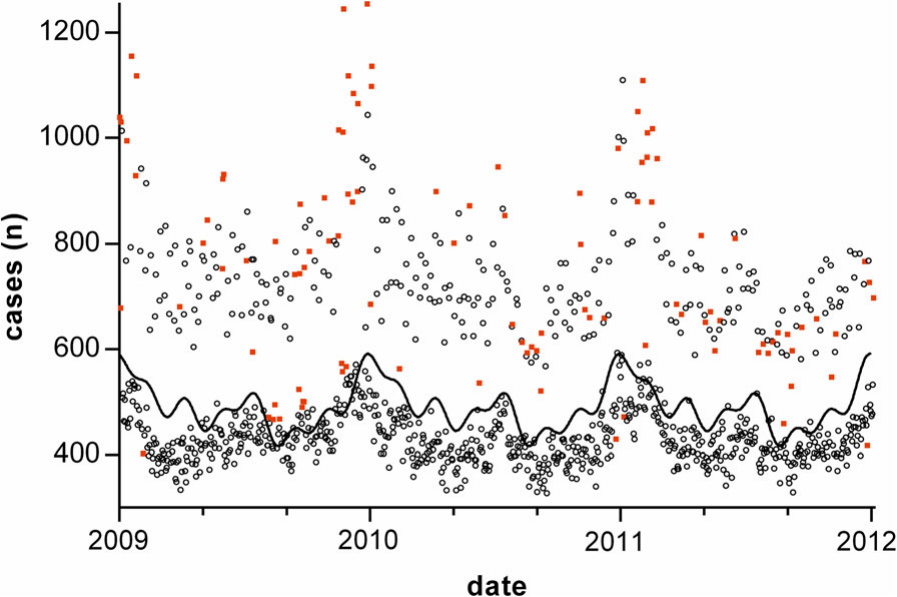
Number of cases observed. *Circles* and *red square* are number of cases observed each day. A *circle* represents a day correctly predicted (close to 100 cases) by the model. A *red square* represents a day with incorrect prediction. The curve represents the number of cases which would have been predicted by the periodic function only

**Fig. 3:**
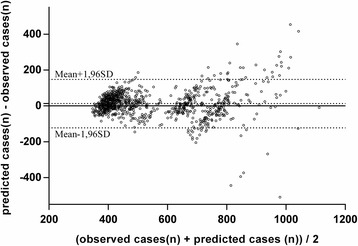
Agreement between number of cases predicted and number observed (test period, Bland and Altman method). The average difference shows if one of our two methods of measurement tends to produce consistently lower or higher values than the other (Predicted number of cases tends to be lower than observed number, here the mean bias is − 13 cases). 95% of the differences between each pair of points are between Mean + 1.96SD and Mean - 1.96 SD (here 95% of the differences between predicted number of cases and observed number were comprised between − 124 and + 150 which are the “limits of agreement”)

For 2009–2011 (1095 days, Fig. 2), the model predicted the number of cases within ± 100 for 991 days (90.5%), with a mean error of −13 cases (95% CI, −17 to −8). Presenting the reliability of the predictions on a Bland and Altman graph (Fig. 3) showed errors greater than 2 SD mainly on days with high activity. Thirty-nine (3.6%) days were predicted with an excess (by more than 100 cases), and 68 (6.2%) were under-estimated. Five hundred seventy nine days with low and high activity were defined through the thresholds of 407 and 607 cases (mean observed ± 100). The model predicted 91% of these days with unusual activity. The mean deviation of 9% wrongly predicted days was 37 cases (95% CI, 29 to 44). The 2 days with greatest underestimates of activity were Sunday Nov. 22, 2009 (788 cases predicted, 1242 observed) and Saturday Dec. 26, 2009 (834 cases predicted, 1251 observed). These were the only 2 days with an error exceeding 350 cases. In comparison, the simpler model, that takes into account the annual average (previous year) of the day of the week increased by a factor of 15.6%, applied to the validation years (2009-2011), showed an accuracy of 54.6% (within ±100), with a mean difference of − 74.72 and 89.9% of underestimated error (> − 100).

## Discussion

To our knowledge no reliable model exists that can be used routinely to predict the daily activity of emergency call centers. Our objective was to construct a dynamic model that was based not only on the case volume of the preceding periods [26, 27] or on sporadic factors. Two techniques, either linear regression models including calendar variables or time series models, are generally used separately for forecasting the daily number of emergency department visits [28]. Our model, based on both calendar and epidemiological variables and including a periodic function to account for the interest of time series, combines the benefit of several types of analysis. Using only variables available at least the week preceding the prediction period, we were able to obtain good results for the prediction of the center’s daily case volume. Application of the model to the period not used for its construction (in order to avoid model optimism) provided a prediction within ± 100 cases for 90.5% of the days studied. With the simpler prediction method that takes into account only the annual average (previous year) of the day of the week and the annual upwards trend, the accuracy appeared quite poor compared to our more complex model. This result is interesting but not surprising since this method does not take into account the season, public and school holidays and seasonal epidemic factors. Even if the prediction of activity peaks was less reliable, in than 90% of the days with unusual activity (out of a priori defined limits: mean case volume ± 100) were predicted correctly, and only 2 exceptional days were underestimated with an error exceeding 350 cases. This less accurate precision in peaks of activity may stem from the numerous factors that were not taken into account, often difficult to predict and unreproducible (such as a worldwide unseasonal flu epidemic, a catastrophe, or a large gathering) and therefore impossible to use in this type of model. These two greatly underestimated days corresponded to 2 winter weekends in the exceptional context of the A(H1N1) flu pandemic [29–32], and the controversy in France over the influenza vaccination that had led to the population reacting to any symptom appearing following a flu vaccination. Our data were probably inadequate for predicting the high volumes in the setting of the A(H1N1) flu pandemic. No other exceptional factor or catastrophe was identified for these 2 days, which in addition corresponded to records in activity during the period studied.

There were few days with overestimations (3.6%), which would have resulted in the mobilization of excessive personnel. The model’s errors by default would have resulted in a work overload 6.2% of the time, i.e. a deficit of employees on these days. For most of the time, the prediction would make it possible to better distribute the workload by modulating the number of personnel treating calls. The predicted variations would have allowed human resources to be adapted by more or less one operator per day. A prospective study could inform on the impact of this type of organization on the response quality indicators of emergency call centers (e.g., reduction of speed of answer, percentage of calls answered within 60 s). These indicators are inspired by commercial hotlines and are correlated with the number of calls in a day when they are studied with a set organization of personnel. For example, it is known that a high level of calls is associated with an increase in the mean time to ambulance intervention [33].

During the period the model was under construction, the direct interpretation of the relation between the variables showed how important they were in the variability of the call center case volume, but this should be interpreted with precaution. For example, the absence of a significant relation between the winter vacation and the center’s case volume, despite covering a large number of ski resorts, may be related to the presence of the periodic function that takes into account the level of winter activity. These simple data concerning notably the effect of the days of the week corrected by the main known confounding factors have not been published to date. These confounding factors are currently taken into account empirically in the organization of call centers based on the mean activity level observed in each center and projected on future activity in a fixed manner. The approach undertaken herein is original in that it provides an estimation of the effect of each day and makes it possible to take into account the additional effect of other factors. We used a linear generalized additive model (GAM) [21–23] instead of a simple linear model in order to enable other centers the possibility to use nonlinear links in their model, nevertheless all variables used in our construction where considered with linear effect.

### Limitations

The results of this study are based on a model developed and calibrated on a single call center covering a large area (1.8% of the French population). All cases in the geographic zone studied are directed to this single call center. In addition, this area includes urban, periurban, and semirural populations, with major tourist areas creating demographic variations during the year. To use this model in another center, calibration on a sample of years would be necessary to create the center’s periodic function and the case levels. Depending on the economic profile of the area it is possible that other important variables should be taken into consideration. Moreover, it cannot be excluded that the effect associated with explanatory factors change over time, thus regularly requiring a new calibration. We believe that this method can be used elsewhere, including in other countries concerned with adapting to variations in the demand for emergency call services.

The activity of the call center appears higher on weekends, even outside periods of tourist influx. We interpret this as due to the unavailability of primary care practitioners, forcing patients to seek telephone counseling or help in obtaining a medical consultation when most doctors’ practices are closed. The fluctuation attributable to mountain sports is mainly visible over whole weeks, and is largely taken into account by the seasonal variations. Thus over-activity at the weekend is not likely to be related to our geographic specificity.

A potentially more precise model could be considered by adding variables such as air temperature, air pollution, recurrent cultural or sports events, or other epidemics. Indeed, if these factors are recurrent and their effect is stable over time, it should be possible to calibrate the model by taking them into account. However, a model taking a very large number of variables into account would be difficult to calibrate and would not respect the parsimony principle that it should be usable in routine practice and comprehensible.

Predicting the number of ambulance dispatches is more difficult and would be less efficient. Indeed, the prediction of the volume of cases is influenced only by the number of calls, the demand. The prediction of ambulance activity is influenced by demand and also by the medical resources (i.e. the number of ambulances available). In our data a weak correlation was observed between the number of cases per day and the number of interventions. The complexity of predictions based on the knowledge of both the demand and the available resources has already been well described in Intensive Care Units [34]. Finally, even in low-activity periods, maintaining a high intervention capacity is necessary in case of heavy demands arriving simultaneously. Furthermore, these emergency response teams are distributed over the geographic area and to modify their numbers would result in losing a part of the area’s coverage and increasing the time to intervention.

## Conclusion

We have developed a model to predict the case volume of an emergency call center with satisfactory reliability. More than 90% of the days were predicted satisfactorily, using 17 variables available 1 week in advance (seasonality, long-term trends, days of the week, holidays and the regional incidence rates for influenza and gastroenteritis). The model described here could be used to explore other factors that may explain part of the observed increase in the activity of emergency call centers, since it contains the main confounding factors that should be taken into account in assessing the impact on activity. Relying on such predictive models could allow better scheduling of dispatch center staff to match variations in emergency call center volume.

## Additional files

Additional file 1: Control for seasonality, Periodogram and periodic function. (DOCX 63 kb)

Additional file 2: SAS macro used to calibrate the predictive model. (DOCX 15 kb)

Additional file 3: Calibrated predictive model. (DOCX 14 kb)

## Abbreviations

GAM: Generalized additive linear model
SAMU: Service d’Aide Médicale d’Urgence (Mobile emergency and resuscitation unit)
