# Supplementary material for: Daily volume of cases in emergency call centers: construction and validation of a predictive model
Source: Scand J Trauma Resusc Emerg Med. 2017 Aug 29;25:86. doi: 10.1186/s13049-017-0430-9 (PMC5576313; doi:10.1186/s13049-017-0430-9)

**Additional file 1: Control for seasonality**, **Periodogram and Periodic functions**

Seasonality was controlled for by creating sinusoidal terms (breakdown of data into Fourier frequency spectrum) and using the highest frequencies of the signal, making a repetition or “structure” representing the activity’s seasonality.

The spectrum was broken down using the “proc spectra” procedure in SAS 9 software (SAS Institute Inc., Cary, NC, USA), distributed into frequencies from 0 to Pi (Figure A).

**Figure A: Periodogram:**


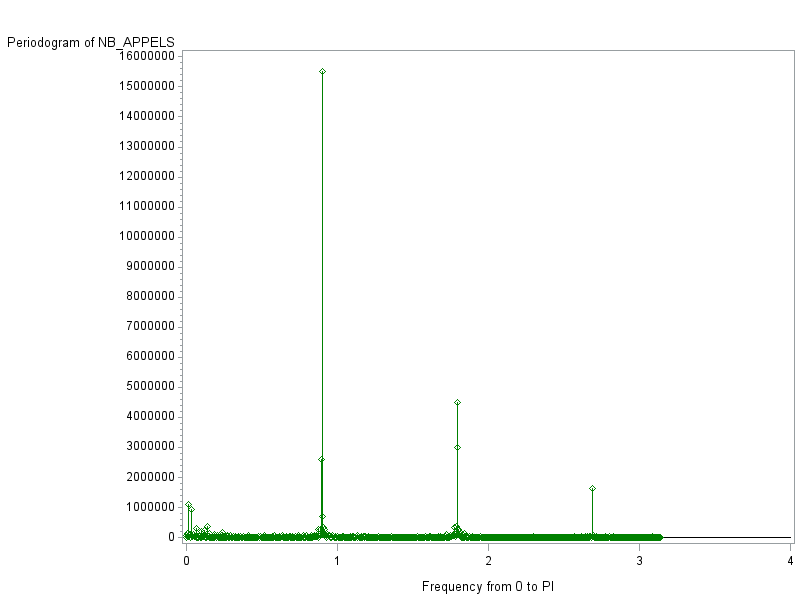


On the graph of the data breakdown, highly present frequencies are noted, around 0.9 Pi, 1.8 Pi, and 2.7 Pi, corresponding to 7-day periods and representing weekly periodicity, which is taken into account by a day of the week variable. Around 0 to 0.2 Pi, other frequencies are present but are much lower.

The precise frequencies and the amount of cases (signal) are shown in Table A (selection of 2-day to 1-year periods). The 365.25-day, 182.625-day, 91.313-day, and 52.179-day periods represent “seasonal” variations longer than 1 month that are present in the data.

| **Table A: Breakdown of the “number of calls” signal per frequency (selection)** | | | |
| --- | --- | --- | --- |
| **Frequency of interest** | | **Signal level** | |
| **Frequency** | **Period (days)** | **Cosinus** | **Sinus** |
| 0.01720 | 365.250 | 1113106.33 | 25355.82 |
| 0.03440 | 182.625 | 94684.04 | 21982.36 |
| 0.06881 | 91.313 | 295518.47 | 6510.38 |
| 0.12042 | 52.179 | 224694.18 | 6891.26 |
| 0.13762 | 45.656 | 353191.49 | 8607.37 |
| 0.89453 | 7.024 | 2592673.61 | 252761.41 |
| 0.89883 | 6.990 | 15506618.58 | 296377.89 |
| 1.79335 | 3.504 | 4491935.01 | 120434.49 |
| 2.69218 | 2.334 | 1639919.57 | 28172.53 |

A composition of these frequencies makes it possible to predict a level related to time using a function of this type:

*F(t)= α sin (2π t /365.25)+β cos(2π t /365.25)+ Ω sin (2π t /182.625)+ Є cos(2π t /182.625)+ (…)*

where **α, β, Ω, Є (…)** are determined (separately) by the linear regression coefficients in a model (Table B) which includes only the year as another variable (to cancel the trend).

This model was constructed using the “PROC GAM” procedure in SAS software.

| **Table B: Estimation of the coefficients of the periodic function** | | | | | |
| --- | --- | --- | --- | --- | --- |
|  | **Estimate** | **Standard deviation** | ***t*-value** | ***p*-value** | **Period (days)** |
| **Intercept** | -13027 | 8065.85736 | −1.62 | .1065 |  |
| **Sinus 1** | 17.53805 | 6.35770 | 2.76 | .0059 | 365.25 |
| **Sinus 2** | 1.18322 | 6.37419 | 0.19 | .8528 | 182.625 |
| **Sinus 3** | 5.99192 | 6.37232 | 0.94 | .3472 | 91.13 |
| **Sinus 4** | −15.05307 | 6.36204 | −2.37 | .0181 | 52.179 |
| **Cosinus 1** | 34.85995 | 6.30855 | 5.53 | <.0001 | 365.25 |
| **Cosinus 2** | 35.96897 | 6.30428 | 5.71 | <.0001 | 182.625 |
| **Cosinus 3** | 19.18498 | 6.35446 | 3.02 | .0026 | 91.313 |
| **Cosinus 4** | 8.98752 | 6.36995 | 1.41 | .1585 | 52.179 |

The sinusoidal function (graphically represented by Figure B) can be constructed based on the four periods selected to represent seasonality; periods greater than 1 month but less than 1 year:

*F(t)= 17.53805 sin(2π t /365.25)+ 34.85995 cos(2π t /365.25)
+ 1.18322 sin(2π t /182.625)+ 35.96897 cos(2π t /182.625)*

*+ 5.99192 sin(2π t /91.313) + 19.18498 cos(2π t /91.313)*

*-15.05307 sin(2π t /52.179) + 8.98752 cos(2π t /52.179)*

**Figure B: Graph of the sinusoidal function over the calibration period, from January 2005 to December 2009**


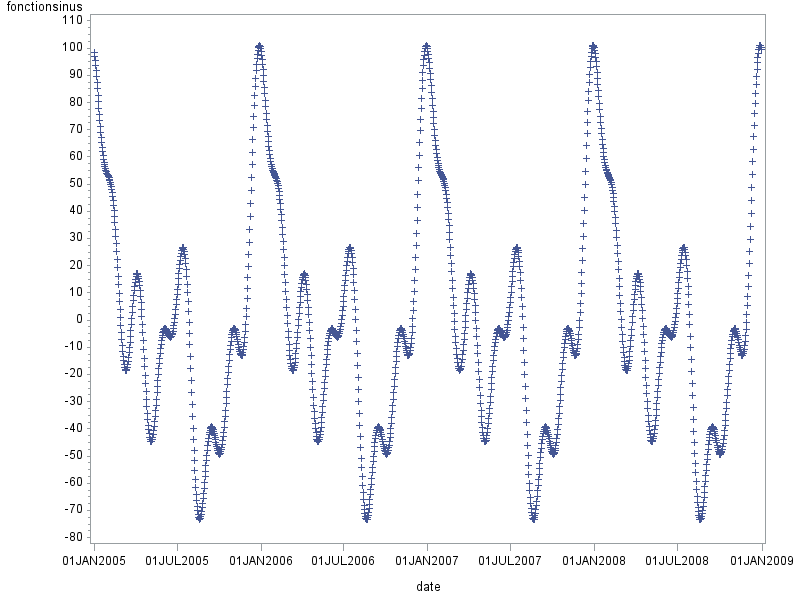

Supplement: Supplementary file 1 — Control for seasonality, Periodogram and periodic function. (DOCX 63 kb) [file 13049_2017_430_MOESM1_ESM.docx]
