# Supplementary material for: Daily volume of cases in emergency call centers: construction and validation of a predictive model
Source: Scand J Trauma Resusc Emerg Med. 2017 Aug 29;25:86. doi: 10.1186/s13049-017-0430-9 (PMC5576313; doi:10.1186/s13049-017-0430-9)
Supplement: Supplementary file 2 — SAS macro used to calibrate the predictive model. (DOCX 15 kb) [file 13049_2017_430_MOESM2_ESM.docx]

**Additional file 2: SAS macro used to calibrate the predictive model**

**data** Table ; set Table ; PI=**4***atan(**1**);

fonctionsinus=**17.53805***sin(**2***PI*date/**365.25**)+ **34.85995***cos(**2***PI*date/**365.25**)

+ **1.18322***sin(**2***PI*date/**182.625**)+ **35.96897***cos(**2***PI*date/**182.625**)

+ **5.99192***sin(**2***PI*date/**91.313**) + **19.18498***cos(**2***PI*date/**91.313**)

-**15.05307***sin(**2***PI*date/**52.179**) + **8.98752***cos(**2***PI*date/**52.179**) ; **run** ;

**proc** **gam** data=Table plots=components(commonaxes clm);

model n_calls =

param(fonctionsinus)

param(year)

param(monday)

param(tuesday)

param(wednesday)

param(thursday)

param(friday)

param(sunday)

param(public_holiday)

param(Christmas_vacation)

param(winter_vacation)

param(autumn_vacation)

param(summer_vacation)

param(spring_break_vacation)

param(winter_vacation_paris)

param(influenza_incidence_rate)

param(gastroenteritis_incidence_rate)

/ dist=normal anodev=refit /*method=gcv*/;

output out=Table_pred1 pred=predit;

**run**;
