# Supplementary material for: Daily volume of cases in emergency call centers: construction and validation of a predictive model
Source: Scand J Trauma Resusc Emerg Med. 2017 Aug 29;25:86. doi: 10.1186/s13049-017-0430-9 (PMC5576313; doi:10.1186/s13049-017-0430-9)
Supplement: Supplementary file 3 — Calibrated predictive model. (DOCX 14 kb) [file 13049_2017_430_MOESM3_ESM.docx]

**Additional file 3: Calibrated predictive model**

fonctionsinus=**17.53805***sin(**2***PI*date/**365.25**)+**34.85995***cos(**2***PI*date/**365.25**)

+ **1.18322***sin(**2***PI*date/**182.625**)+ **35.96897***cos(**2***PI*date/**182.625**)

+ **5.99192***sin(**2***PI*date/**91.313**) + **19.18498***cos(**2***PI*date/**91.313**)

-**15.05307***sin(**2***PI*date/**52.179**) + **8.98752***cos(**2***PI*date/**52.179**)

F(t)= -**13876**

+ **0.55046***fonctionsinus

+ **7.10339***year

+ **376.07414***monday

-**3.01982***tuesday

-**14.62459***wednesday

-**5.74918***thursday

+**1.96028***friday

+**251.74887***sunday

+**89.05141***christmas_vacation

+**12.09922***winter_vacation

-**3.80929***autumn_vacation

-**4.44251***summer_vacation

-**15.82818***spring_break_vacation

-**8.23398*** winter_vacation_paris

+**0.05764***influenza_incidence_rate

+**0.04740***gastroenteritis_incidence_rate

+**295.02641***public_holiday
